# Three different Plasmodium species show similar patterns of clinical tolerance of malaria infection

**DOI:** 10.1186/1475-2875-8-158

**Published:** 2009-07-14

**Authors:** Ivo Müller, Blaise Genton, Lawrence Rare, Benson Kiniboro, Will Kastens, Peter Zimmerman, James Kazura, Michael Alpers, Thomas A Smith

**Affiliations:** 1 Department of Public Health & Epidemiology, Swiss Tropical Institute, Socinstrasse 57, Postfach CH-4002, Basel, Switzerland; 2 Papua New Guinea Institute of Medical Research, PO Box 60, Goroka, Papua New Guinea; 3 Center for Global Health & Diseases, Case Western Reserve University, Cleveland, OH, USA

## Abstract

**Background:**

In areas where malaria endemicity is high, many people harbour blood stage parasites without acute febrile illness, complicating the estimation of disease burden from infection data. For *Plasmodium falciparum *the density of parasitaemia that can be tolerated is low in the youngest children, but reaches a maximum in the age groups at highest risk of infection. There is little data on the age dependence of tolerance in other species of human malaria.

**Methods:**

Parasite densities measured in 24,386 presumptive malaria cases at two local health centres in the Wosera area of Papua New Guinea were compared with the distributions of parasite densities recorded in community surveys in the same area. We then analyse the proportions of cases attributable to each of *Plasmodium falciparum*, *P. vivax*, and *P. malariae *as functions of parasite density and age using a latent class model. These attributable fractions are then used to compute the incidence of attributable disease.

**Results:**

Overall 33.3%, 6.1%, and 0.1% of the presumptive cases were attributable to *P. falciparum*, *P. vivax*, and *P. malariae *respectively. The incidence of attributable disease and parasite density broadly follow similar age patterns. The logarithm of the incidence of acute illness is approximately proportion to the logarithm of the parasite density for all three malaria species, with little age variation in the relationship for *P. vivax or P. malariae*. *P. falciparum *shows more age variation in disease incidence at given levels of parasitaemia than the other species.

**Conclusion:**

The similarities between Plasmodium species in the relationships between parasite density and risk of attributable disease are compatible with the hypothesis that pan-specific mechanisms may regulate tolerance to different human Plasmodia. A straightforward mathematical expression might be used to project disease burden from parasite density distributions assessed in community-based parasitological surveys.

## Background

Residents of malaria endemic areas frequently harbour asexual blood stage parasites without developing symptoms or signs of acute febrile illness, implying that some degree of clinical tolerance to parasitaemia is acquired through repeated exposure to and experience with chronic blood stage infection. Epidemiological studies of this phenomenon have focused mainly on *Plasmodium falciparum*, the dominant malaria species world wide, and attempted to quantify this complex clinical phenotype at a population level by estimating the peripheral parasite density at which body temperature exceeds a specific level or cut off value, i.e. the pyrogenic threshold [1]) or the probability of acute febrile illness as a function of parasite density [2-4]. In contrast, similar analyses of clinical tolerance to other major malaria species that infect humans, *Plasmodium vivax*, *Plasmodium malariae *and *Plasmodium ovale*, are limited to one study of *P. vivax *from Punjab [5] and another of *P. ovale *from Senegal [6].

Understanding the differences in clinical tolerance to parasitaemia among various malaria species may be important in areas of the world where *P. vivax *and *P. falciparum *are co-endemic such as in Asia, the Pacific and South America. Anti-malarial drug resistance or deployment of vaccines that preferentially affects one species may alter innate and adaptive immunity and clinical tolerance to the other.

In highly endemic areas for *P. falciparum*, the fever threshold expressed in terms of the density of parasitaemia in peripheral blood at which a given body temperature is exceeded declines progressively after the age of one year [1,2,7]. Thus, children with high parasite densities tend to be asymptomatic compared with adults or adolescents with similar levels of peripheral parasitaemia. Nevertheless adults have a lower incidence of clinical malaria attacks than children. because *P. falciparum *density is on average controlled at a lower level in adults than children

This is consistent with the idea that tolerance is the consequence of an immunological response with little memory, stimulated by toxins released during schizogony, and several possible mediators of tolerance have been proposed with this in mind, notably the anti-inflammatory molecule nitric oxide (NO) [8] and antibodies to GPI [1]. However recent studies in Papua New Guinea suggest that cytokine responses to GPI can better account for both immunological and epidemiological patterns [9].

Variations in tolerance are only one possible explanation for differences in the operating characteristics of diagnostic thresholds of peripheral parasitaemia in *P. falciparum*. Changes in pyrogenic threshold could also be explained in terms of differences in the ratio of circulating to sequestered parasites. Pyrogens or putative malaria toxins are released when sequestered *P. falciparum*-infected erythrocytes burst during schizogony. Hence pyrogen concentrations may reflect more directly the density of sequestered parasites than that of trophozoites in the peripheral circulation. At present, approaches for assessing the number of sequestered *P. falciparum *parasites in the living human host remain controversial [10,11], and there is no means of reliably quantifying circulating toxix(s) until their molecular nature is better understood. In contrast, the rate of schizogony and level of malaria toxin release should be approximately proportional to the peripheral blood parasite density in *P. vivax *and *P. malariae *infection since these species are not thought to sequester in deep vascular beds.

In population based studies, it is possible to estimate the apparent degree of clinical tolerance relative to the probability of an individual experiencing a given peripheral density. This approach may be a better way of assessing tolerance than using specific diagnostic cut-off values since the latter depends on the extent of incidental parasitaemia in a population as well as the pathogenic effect of a given parasite density. We have carried out an analysis of the relationships between the incidence of acute illness attributable to *P. vivax *and *P. malariae *and the densities of circulating parasites in an area of Papua New Guinea where these two malaria species as well as *P. falciparum *are highly endemic, over the period 1991–2003. The results for *P. vivax *and *P. malariae *are compared with those for *P. falciparum*, with consideration of the age dependence of apparent tolerance for each malaria species and implications for models of malaria pathogenesis and disease burden.

## Methods

### Study area and population

The study involved residents of 29 villages in the Wosera area of East Sepik Province, Papua New Guinea. The average population over the study period was 11,627 persons. Transmission is perennial with estimated inoculation rates for *P. falciparum*, *P. vivax *and *P. malariae *averaging 35, 12, and 10 infectious bites per annum respectively during the period of 1990–1992 [12].

A detailed description of malaria species infection rates has been presented elsewhere [13,14]. Malariometric surveys showed an overall decrease in overall *Plasmodium *spp. prevalence rate from 60% in the early 1990s to 35% by 2002. The reduction was from 38% to 22% for *P. falciparum*, 20% to 10% for *P. vivax*, and 16% to 4% for *P. malariae *[15]. This was probably related to gradual increase in the use of insecticide-treated nets and change in the national policy for anti-malarial treatment of asymptomatic parasitaemia.

### Case detection and investigation

The area is serviced by two local health centers located in the villages of Kunjingini and Kaugia and the health records of 24,386 presumptive malaria cases diagnosed at these health centres during the study period. A presumptive case of malaria was defined as an outpatient with a clinical diagnosis of malaria made by nurses staffing one or other of these health centers. The diagnostic procedures used followed the guidelines of the Papua New Guinea Department of Health [16], and usually the diagnosis of malaria was based on a history of fever without obvious symptoms or signs of another disease [17]. Malaria was the most frequent clinical diagnosis, followed by acute respiratory infections and skin conditions [18].

Treatment procedures at the health centres also followed the guidelines of the PNG Department of Health, which recommended malaria treatment for all patients with fever [16] with various changes in drug regimens during the course of the study. All individuals with a presumptive diagnosis of malaria had a blood film prepared to detect malaria parasites.

A research nurse from the Papua New Guinea Institute of Medical Research attended the outpatient clinics from 8 AM to 2 PM Monday through Friday and further investigated all presumptive malaria cases reporting on those days. After recording demographic information a pertinent history of illness was taken, e.g. duration of self-appraised fever before reporting to the health center, a standardized physical examination that included auscultation of the chest and heart and abdominal palpation to detect pain and enlargement of the spleen and liver was performed followed by a finger prick blood sample collected for microscopic detection of malaria by microscopy and haemoglobin measurement (for details see [14,19]).

### Cross-sectional surveys

Blood films from control subjects were collected in three series of cross-sectional community surveys conducted in 1991/92 [13], 1998/99 [20] and 2001–03[15]. Only parasitological data from participants, who had no signs of concurrent febrile illness and did not report illness during the previous week were included. Blood film results from 31,455 participants were included in the analysis of the controls.

### Laboratory examination for malaria

Blood films were stained with 4% Giemsa; 100 microscopic thick film fields were inspected before a slide was being declared malaria-negative. For malaria-positive blood films, parasite species were identified and densities recorded as the number of parasites per 200 WBC. This procedure was followed for each species. Densities were converted to asexual parasites per *μ*l of blood assuming 8000 WBC per *μ*l. Routine quality control procedures were performed [19]. Briefly, a 10% random sample of slides and all those that had a density between one and five asexual stage *P. falciparum*, *P. vivax*, *P. malariae*, or *P. ovale *were re-read by a supervisor microscopist blinded to the first reading. When the results on a batch of ~1,000 slides did not reach 75% agreement on positivity/negativity, species and density (including a margin of error that increased with increasing density), the entire batch was re-read and the same quality control applied again. Clinical cases observed to be infected with more than one malaria species were included only in the analyses of the dominant species on the assumption that this was the most likely to be the cause of the febrile illness.

### Model for dependence of fever risk on parasite density

Cases corresponded to the presumptive malaria cases detected by passive case detection of persons reporting to local health centers plus a positive blood smear for malaria. Control slides were collected from asymptomatic individuals who donated during the cross-sectional surveys. Our analysis resolved the distribution of parasite densities in the presumptive malaria cases into two components, corresponding to non-malaria illness and to episodes of clinical malaria.

**Figure 1:**
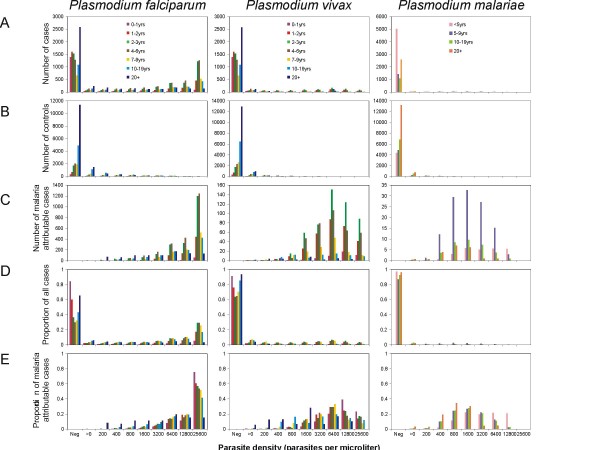
**Age-, species-, and parasite density-specific data and results**. A: numbers of cases included in the analysis. B: numbers of controls included in the analysis. C: estimated numbers of malaria attributable cases. D: proportions of all cases. E: proportions of malaria specific cases.

The parasite densities of controls and cases were divided into *K *ordered categories, *k *= 1, 2,.., *K*. We define *n*(*k*) to be the number of presumptive malaria cases in parasite density category *k*, and *θ*_*s*_(*k*) to be the corresponding proportion of all presumptive cases (Figure 1a). We define *θ*_*c*_(*k*) to be the proportion of control individuals in category *k *(Figure 1b), and *θ*_*m*_(*k*) to be the proportion of true clinical malaria patients in the same category (Figure 1e). *θ*_*s*_(*k*) then arises as the mixture:

(1)

on the assumption that *θ*_*c*_(*k*) also gives the distribution of parasite densities in those presumptive cases that in reality have non-malaria aetiology, and where *Λ *is the overall proportion of cases whose illness is attributable to malaria (the malaria-attributable fraction). A latent class model was then fitted to the counts of cases and controls, using the simulation-based Bayesian approach described in detail previously[21], implemented in WinBUGS [22] (code available at )., This provided estimates both of *Λ *and of *λ *(*k*), the mixing proportion among the cases in each category *k*, where:

(2)

subject to the constraint that *λ *(*k*) is an increasing function of *k *[21]. These were then used to obtain estimates of the number of malaria attributable cases in the category, *λ *(*k*)*n*(*k*) (Figure 1c). The effects of age and *Plasmodium *species were summarized by carrying out this analysis separately for each age group of host and each of the three malaria species considered.

### Operating characteristics of case definitions

In epidemiological studies, clinical malaria is frequently defined to correspond to all febrile episodes with parasite densities exceeding a given cut-off value (e.g. [23-25]). Following [26], the sensitivity of the cut-off that corresponds to the lower bound of category *C*, is given by:

(3)

the positive predictive value by:

(4)

and the specificity is:

(5)

### Calculation of clinical malaria incidence

To compare malaria incidence between species and age groups at different parasite densities we estimated the approximate person-time at risk in each category, using population sizes and age distributions from the Wosera demographic surveillance system [27] and the distributions of parasite densities in the control samples. The overall incidence of clinical malaria in each parasite density class was then computed by dividing the total number of episodes by this overall person-time-at-risk. The estimated incidence was then re-scaled to give an adjusted overall incidence of presumptive malaria attending health facilities equal to our previously published estimate of 0.49 episodes per person-year [18] for outpatient visits to Kunjingini.

## Results

### Age patterns of infection

**Figure 2:**
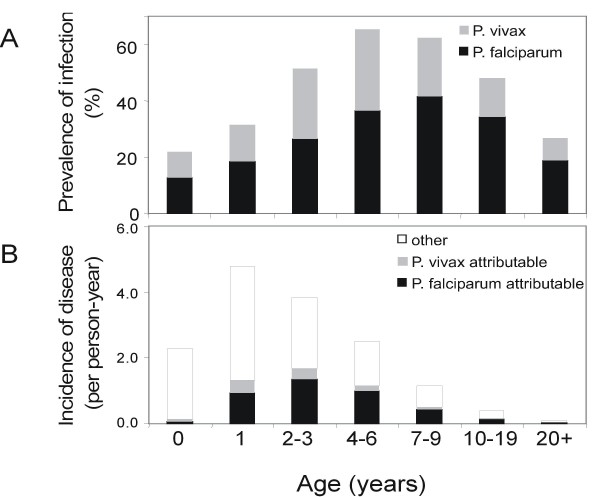
**Prevalence of infection and incidence of disease by age**. A: prevalence of infection in controls. B: Incidence of presumptive and attributable malaria cases.

27.3% of blood smears from control subjects were positive by blood slide for *P. falciparum*, 14.1% for *P. vivax*, 7.0% for *P. malariae*, and nil for *P. ovale*. 5.5% of slides were positive for more than one species. Infection reached a peak prevalence in children between four and six years of age (*P. vivax*) and between seven and nine years of age (*P. falciparum*) (Figure 2)) [12,13,15]. *Plasmodium malariae *infection is much less frequent than either of the other two species and reaches a maximum prevalence of 10.3% in the 7–9 year age group. Average parasite densities in infected persons are also strongly age dependent in both cases and controls and peak at lower ages than the prevalence of infection (Table 1).

**Table 1:** Parasites densities in clinical cases and community controls

	**CASES**		**CONTROLS**	
		
	**Median**	**IQR**	**Median**	**IQR**
***P. falciparum***				
<1	9,480	[640, 39,800]	3000	[740, 14,800]
1	19,880	[4,640, >40,000]	1,200	[160, 5,360]
2–3	21,100	[5,600, >40,000]	1,600	[320, 6,550]
4–6	17,220	[3,520, >40,000]	680	[200, 2,560]
7–9	14,840	[2,440, >40,000]	480	[160, 1,680]
10–19	9,080	[1,100, 31,460]	240	[80, 720]
20+	1,600	[320, 9,610]	160	[80, 440]
				
***P. vivax***				
<1	4,480	[240, 15,260]	120	[70, 1,520]
1	5,540	[1,360, 12,870]	480	[130, 2,960]
2–3	4,500	[920, 12,820]	440	[120, 1,600]
4–6	2,440	[320, 9,120]	200	[120, 640]
7–9	1,280	[200, 7,480]	160	[80, 280]
10–19	300	[80, 3,380]	80	[40, 200]
20+	120	[80, 280]	80	[40, 160]
				
***P. malariae***				
<1	220	[90, 350]	80	[40, 740]
1	420	[120, 1,910]	200	[40, 360]
2–3	1380	[210, 3,360]	320	[120, 800]
4–6	1160	[450, 3,240]	320	[120, 680]
7–9	960	[280, 2,400]	160	[80, 360]
10–19	440	[120, 1760]	120	[60, 200]
20+	120	[80, 480]	80	[40, 160]

### Age patterns of presumptive malaria episodes and attributable fractions

The incidence of presumptive malaria morbidity is highest in younger children, peaking in one year-old children (Figure 2b). The latent class model provides estimates of the proportions of these clinical attacks attributable to each of the three malaria species, in each age group, and parasite density class (Figure 1c to 1e).

**Figure 3:**
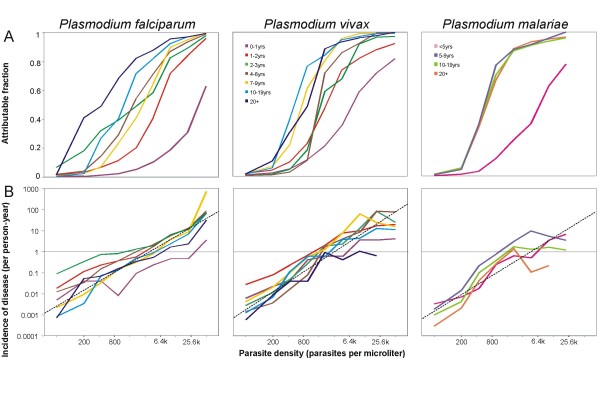
**Attributable fractions and incidence of disease**. A: attributable fractions by age, species and parasite density. B: incidence of attributable illness by age, species and parasite density. Dashed lines correspond to the regressions given in Table 2 with the line for *P. falciparum *corresponding to the analysis that excludes children < 1 year old.

Figure 3a gives values of *λ *(*k*) for each of the *Plasmodium *species, host age groups, and parasite density categories. For each species, the youngest age group has the lowest attributable fraction at any given density (Figure 3a). For *P. vivax *and *P. malariae*, there is a very steep increase in the attributable fraction with parasite density at around 800 parasites per *μ*l for all age groups except the youngest, with little age variation in the curve. *Plasmodium falciparum *shows a greater difference between age groups in attributable fractions at a given level of parasitaemia, with a general tendency (as previously reported [2]) for the older age groups to have higher attributable fractions at any given parasite density.

### Operating characteristics of case definitions

**Figure 4:**
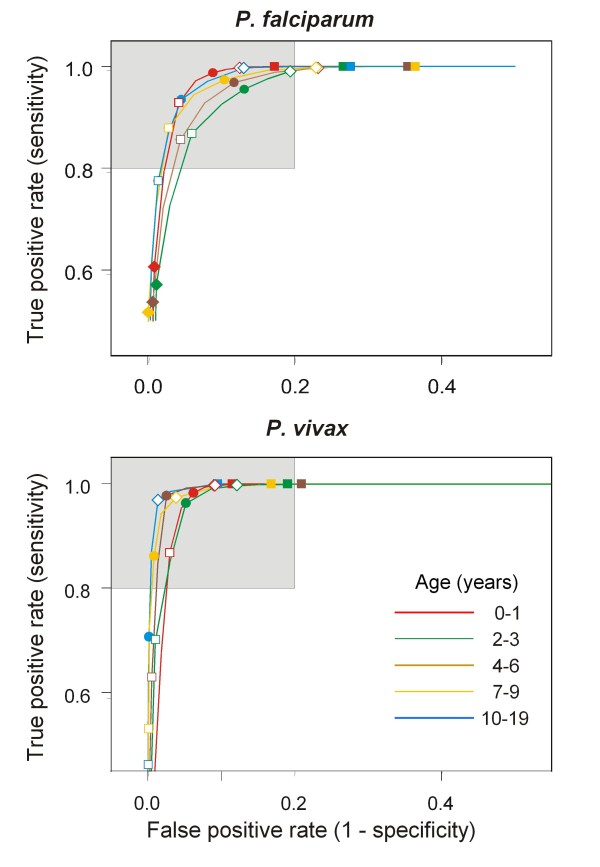
**Estimated operating characteristics of cutoffs**. Receiver Operating Characteristic (ROC) curves of density cut-offs for the definition of *P. falciparum *and *P. vivax *clinical episodes. Shaded area: Sensitivity and Specificity > 80%. Density cutoffs: filled squares: 200/*μ*l; open diamonds: 800/*μ*l; filled circles: 3,200/*μ*l; open squares 12,800/*μ*l; filled diamonds: 40,000/*μ*l.

The estimates of the sensitivity of diagnostic cut-offs by age and parasite density show the inverse pattern to the corresponding attributable fractions (Figure 4 and Additional file 1), indicating that a low cut-off is needed to ensure high sensitivity in older individuals, while in younger children, a higher cut-off can be used. The patterns are broadly similar for all three species. A high specificity is achieved, even with very low cut-offs for all age groups, and for all species, but particularly for *P. malariae *in which the presence of any patent parasitaemia in a clinical case has an estimated specificity of over 90% in all age groups.

### Age- and parasite density patterns of attributable disease

The estimates of incidence of attributable disease by age and parasite density (Figure 3b) show very different patterns to those for the attributable fractions. *Plasmodium falciparum *does indeed show some age variations (as previously reported[2]), but these are most evident at relatively low parasite densities. At the high parasite densities found in most *P. falciparum*-attributable cases, there is little difference between most of the age groups in incidence of attributable disease (Figure 3b). Children in their first year of life, however, stand out as having lower incidence than other age-groups at relatively high parasite density levels (≥ 800 parasites per *μ*l).

*P. vivax *and *P. malariae *share a different pattern. After adjustment for the time at risk in the different density categories, there seems to be almost no difference by age in parasite density specific incidence of attributable disease in the lower density classes (Figure 3b). At high parasite densities in which the data for these species are sparse, there is more variation between age groups in incidence. In contrast to *P. falciparum*, there is a suggestion of a plateau in incidence as densities increase but this is based on very little data, as high density infections with these species are very unusual. The data for high density *P. vivax *and *P. malariae *are rather sparse.

To translate the parasite density specific values into overall age- and species specific incidence of disease (Figure 2b), we sum both the estimated time at risk and the number of attributable episodes across all parasite density classes. *P. vivax *attributable morbidity peaks in 1 year-olds, and *P. falciparum *morbidity peaks in 2–3 year olds (Figure 2b). *P. malariae *attributable clinical disease is restricted almost entirely to the 5–9 year age group, but even in this age group the incidence of 0.031 episodes per-person-year is small in comparison with that of acute illness attributable to the other species.

A simple empirical relationship between parasite density and incidence of attributable disease seems to approximately hold across most age-groups and all three *Plasmodium *species (ascending straight lines in Figure 3b):

(6)

where *I*_*y *_is the incidence of disease episodes per person-year experienced at parasite density *y *parasites per *μ*l blood. This relationship lends itself to a simple formula for estimating burden of disease from community-based parasite density distributions:

(7)

where *I*_*total *_is the incidence per person-year, which can be calculated separately for each *Plasmodium *species. The estimates of *β*_0 _and *β*_1_obtained for each species by a linear regression through the datapoints in Figure 3 are remarkably similar to each other (Table 2).

**Table 2:** Regression parameters for relationship of incidence of attributable disease with parasites density

	*Intercept β_0_,, [95% CI]*		*Slope, β_1_, [95% CI]*	
*P. falciparum *(excluding < 1 year old)	-4.29	[-4.62, -3.97]	1.33	[1.24, 1.42]
*P. falciparum *(all)	-5.03	[-5.62, -4.43]	1.40	[1.23, 1.57]
*P. vivax*	-5.51	[-6.07, -4.95]	1.57	[1.40, 1.73]
*P. malariae*	-5.75	[-6.75, -4.75]	1.56	[1.26, 1.87]

## Discussion

Previous studies of malaria tolerance by age have generally concentrated on the age patterns in the attributable fractions (Figure 3a). By focusing on the diagnostic performance of different cut-offs and the identification of pyrogenic thresholds, such studies conclude that the age distribution of the pyrogenic threshold is similar to that of parasite densities, with high diagnostic cut-offs required in young children, who thus appear more tolerant. However the diagnostic performance of these cutoffs depend not only on pyrogenic thresholds, but also on age patterns of non-malaria fevers. The present analyses allow for age-variations in overall fever incidence in reporting incidence of attributable disease and suggests different age-patterns of tolerance.

Highly relevant to the mechanism of tolerance is the question of whether it is specific for a given species of *Plasmodium*, or whether it is common to all malaria species. There may even be cross-tolerance with bacteria [9], since malariatherapy studies found that Plasmodium infection can reduce the response to bacterial endotoxins [28,29].

Prior to our study there was little epidemiological evidence of whether tolerance to different *Plasmodium *species follow similar dynamics. In the Punjab, the relationship between morbidity and parasite densities was found to be age-dependent in *P. falciparum*, but not so in *P. vivax *[5]; however, unlike the situation in Papua New Guinea, this was observed in an area of relatively low malaria transmission where repeated infections with different malaria species are infrequent.

In Wosera, there are substantial differences between species in the prevalence and density of infections and in clinical incidence. *P. falciparum *is clearly the most important cause of malaria morbidity (82.9%), followed by *P. vivax *(15.1%), with *P. malariae *accounts for only 2.1% of attributable cases. However, across most densities and age groups the incidence of disease at a given parasite density is similar for all three species, and much of the variation between the lines for different age groups in Figure 3b is in the less frequent density classes (i.e. the low density classes for *P. falciparum*, and the higher ones for *P. vivax *and *P. malariae*), where sampling variation clearly plays a role. For all three species, the lowest attributable fractions at any given parasite density occur in the youngest age group (Figure 3a), but tolerance is achieved with similar age dynamics, even though infection with the different species occurs at different rates, and the age patterns of attributable morbidity are very different (Figures 3 and 2b).

The most important difference in age patterns of morbidity is the relatively high incidence of *P. vivax *morbidity compared with *P. falciparum *in the youngest age groups (Figure 2b). This contrasts with the higher prevalence of *P. falciparum *in the same age groups (Figure 2a) and with the higher entomological inoculation rate of the latter parasite[12]. This seems to mainly reflect better control of *P. falciparum *densities in infants than of *P. vivax *densities, and could reflect better protection for the former by maternal antibodies [30] or/and active sensitization *in utero *[31]. Pregnant women in the Wosera are more likely to be infected with *P. falciparum *than with *P. vivax *[32], which might lead to more acquired protection against high density parasitaemia in the former case. However it also appears to be the case that, at any given peripheral parasite density, *P. falciparum *is less likely to cause disease in infants than it would in older age groups.

Systematic variation in the ratio of circulating to sequestered parasites with age has previously been suggested as explanation for patterns of age- and seasonal variation in apparent tolerance of *P. falciparum *in infants[33]. An important biologic difference between *P. falciparum *and the other species is sequestration of late trophozoites in the former, which means that the density of *P. falciparum *in peripheral blood is an indirect and possibly imprecise measure of the rate of pyrogen release at schizogony. Variation in the ratio of circulating to sequestered parasites presumably contributes imprecision to our analyses and also lends itself as a possible explanation of why there seems to be more age variation in levels of tolerance for *P. falciparum *than for the other species (Figures 3), despite the greater sample size.

The interpretation of such variations also needs to take into consideration the logarithmic scales used on the axes of Figure 3b, so small differences, notably the rather higher incidence at given densities for *P. vivax*, are not very obvious. Even if the mechanisms of tolerance are related, equivalence cannot be assumed in the pyrogenic potential of equal parasite counts of different species, with different biochemistry. The absence of sequestration in *P. vivax *and *P. malariae *means that the rate of schizogony relative to the circulating density must be much lower than for *P. falciparum*, and the longer erythrocytic cycle of *P. malariae *must also mean that it has an even lower rate of schizogony relative to the circulating density.

Variation in age-incidence patterns may also arise because of biases in the available data. Most obviously, not all episodes report to a health facility, so incidence estimates need to be adjusted for imperfect access if they are to be translated into disease burden. Age patterns in illness perception and help-seeking could bias the clinical incidence data, but there is no clear evidence that such biases are important in Wosera. For instance the effect of distance from health facility on help-seeking for febrile illnesses is independent of age group [18]. The control surveys were based on sampling from a complete demographic database representative of the population and so do not represent a substantial source of bias.

Despite the effects of all these other factors, it appears that age variation in clinical incidence mainly arises because of differences in the ability to control parasitaemia, and both age- and species-variation in tolerance are secondary phenomena. Since tolerance may arise in tandem for the different parasite species, this suggests there may be cross-species mechanisms of tolerance, and leads to a similar empirical relationship between parasite density and incidence of attributable disease for all species.

This potentially provides a practical approach for burden of disease assessments in areas with high malaria endemicity, since it could provide a straightforward way of using representative community-based data to avoid the limitations of health management information systems. There is a clear need to evaluate the generalizability of this relationship to other settings, both to evaluate its practical utility for estimating disease burden from survey data, and for further understanding the biology of malaria tolerance.

## Conclusion

In the Wosera area of Papua New Guinea, different human malaria species show similar incidence of attributable disease at the same parasite densities, compatible with the hypotheses that there are pan-specific mechanisms of tolerance. *P. falciparum *shows rather greater differences in apparent tolerance between age groups than *P. vivax *and *P. malariae*, which may in part reflect differences in the ratio of circulating:sequestered parasites, rather than in levels of tolerance. The implication that variations in level of tolerance are of secondary importance in determining overall disease risk and the straightforward mathematical form that can be used to approximate the relationship between parasite density and disease risk, suggest that the latter might be useful for projecting disease burden from parasite density distributions assessed in community-based parasitological surveys.

## Competing interests

The authors declare that they have no competing interests.

## Authors' contributions

IM & TAS designed the study, conducted the analyses and wrote the initial draft of the paper. BG assisted in study design and with TAS and MA established the surveillance of clinical cases. LR, BK, and WK led field work and organised cross-sectional surveys. PZ, JK, MA were responsible for conduct of the 1998/99 and 2001–03 surveys. All authors participated in writing of the manuscript and approved the final version.

## Supplementary Material

Additional file 1 **Operational characteristics of parasite cut-offs**. The additional figure gives age specific values of A: Attributable fractions; B: Sensitivities; C: Specificities for each of the three Plasmodium species. Click here for file

## References

[B1] Rogier C, Commenges D, Trape JF (1996). Evidence for an age-dependent pyrogenic threshold of Plasmodium falciparum parasitemia in highly endemic populations. Am J Trop Med Hyg.

[B2] Smith T, Genton B, Baea K, Gibson N, Taime J, Narara A, Al Yaman F, Beck HP, Hii J, Alpers M (1994). Relationships between Plasmodium falciparum infection and morbidity in a highly endemic area. Parasitology.

[B3] Dicko A, Mantel C, Kouriba B, Sagara I, Thera MA, Doumbia S, Diallo M, Poudiougou B, Diakite M, Doumbo OK (2005). Season, fever prevalence and pyrogenic threshold for malaria disease definition in an endemic area of Mali. Trop Med Int Health.

[B4] Mwangi TW, Ross A, Snow RW, Marsh K (2005). Case definitions of clinical malaria under different transmission conditions in Kilifi district, Kenya. J Infect Dis.

[B5] Prybylski D, Khaliq A, Fox E, Sarwari AR, Strickland GT (1999). Parasite density and malaria morbidity in the Pakistani Punjab. Am J Trop Med Hyg.

[B6] Faye FB, Spiegel A, Tall A, Sokhna C, Fontenille D, Rogier C, Trape JF (2002). Diagnostic criteria and risk factors for Plasmodium ovale malaria. J Infect Dis.

[B7] Miller MJ (1958). Observations on the natural history of malaria in the semi-resistant West African. Trans R Soc Trop Med Hyg.

[B8] Clark IA, Al-Yaman FM, Cowden WB, Rockett KA (1996). Does malarial tolerance, through nitric oxide, explain the low incidence of autoimmune disease in tropical Africa?. Lancet.

[B9] Boutlis CS, Yeo TW, Anstey NM (2006). Malaria tolerance – for whom the cell tolls?. Trends in Parasitology.

[B10] Dondorp AM, Desakorn V, Pongtavornpinyo W, Sahassananda D, Silamut K, Chotivanich K, Newton PN, Pitisuttithum P, Smithyman AM, White NJ, Day NP (2005). Estimation of the total parasite biomass in acute falciparum malaria from plasma PfHRP2. PLoS Medicine.

[B11] Ochola LB, Marsh K, Lowe B, Gal S, Pluschke G, Smith T (2005). Estimation of the sequestered parasite load in severe malaria patients using both host and parasite markers. Parasitology.

[B12] Smith T, Hii JL, Genton B, Muller I, Booth M, Gibson N, Narara A, Alpers MP (2001). Associations of peak shifts in age – prevalence for human malarias with bednet coverage. Trans R Soc Trop Med Hyg.

[B13] Genton B, Al Yaman F, Beck HP, Hii J, Mellor S, Narara A, Gibson N, Smith T, Alpers MP (1995). The epidemiology of malaria in the Wosera area, East Sepik Province, Papua New Guinea, in preparation for vaccine trials. I. Malariometric indices and immunity. Ann Trop Med Parasitol.

[B14] Genton B, Al Yaman F, Beck HP, Hii J, Mellor S, Rare L, Ginny M, Smith T, Alpers MP (1995). The epidemiology of malaria in the Wosera area, East Sepik Province, Papua New Guinea, in preparation for vaccine trials. II. Mortality and morbidity. Ann Trop Med Parasitol.

[B15] Kasehagen LJ, Mueller I, McNamara DT, Bockarie MJ, Kiniboro B, Rare L, Lorry K, Kastens W, Reeder JC, Kazura JW (2006). Changing patterns of Plasmodium blood-stage infections in the Wosera region of Papua New Guinea monitored by light microscopy and high throughput PCR diagnosis. Am J Trop Med Hyg.

[B16] Health PNGDoP (1993). Standard Treatment for Common Illnesses of Children in Papua New Guinea.

[B17] Genton B, Smith T, Baea K, Narara A, Al Yaman F, Beck HP, Hii J, Alpers M (1994). Malaria: how useful are clinical criteria for improving the diagnosis in a highly endemic area?. Trans R Soc Trop Med Hyg.

[B18] Muller I, Smith T, Mellor S, Rare L, Genton B (1998). The effect of distance from home on attendance at a small rural health centre in Papua New Guinea. Int J Epidemiol.

[B19] Genton B, D'Acremont V, Rare L, Baea K, Reeder JC, Alpers M, Muller I (2008). Plasmodium vivax and Misex Infections Are Associated with Severe Malaria in Children: A Prospective Study from Papua New Guinea. PLOS Medicine.

[B20] Kasehagen LJ, Mueller I, Kiniboro B, Bockarie MJ, Reeder JC, Kazura JW, Kastens W, McNamara DT, King CH, Whalen CC (2007). Reduced Plasmodium vivax erythrocyte infection in PNG Duffy-negative heterozygotes. PLoS ONE.

[B21] Vounatsou P, Smith T, Smith AF (1998). Bayesian analysis of two-component mixture distributions applied to estimating malaria attributable fractions. J Roy Stat Soc C-Applied Statistics.

[B22] Spiegelhalter DJ, Thomas A, Best N, Lunn D (2003). Winbugs Version 1.4.

[B23] Trape JF, Peelman P, Morault-Peelman B (1985). Criteria for diagnosing clinical malaria among a semi-immune population exposed to intense and perennial transmission. Trans R Soc Trop Med Hyg.

[B24] Greenwood BM, Bradley AK, Greenwood AM, Byass P, Jammeh K, Marsh K, Tulloch S, Oldfield FS, Hayes R (1987). Mortality and morbidity from malaria among children in a rural area of The Gambia, West Africa. Trans R Soc Trop Med Hyg.

[B25] Snow RW, Lindsay SW, Hayes RJ, Greenwood BM (1988). Permethrin-treated bed nets (mosquito nets) prevent malaria in Gambian children. Trans R Soc Trop Med Hyg.

[B26] Smith T, Schellenberg JA, Hayes R (1994). Attributable fraction estimates and case definitions for malaria in endemic areas. Stat Med.

[B27] NETWORK I (2002). Population, Health and Survival at INDEPTH Sites.

[B28] Heyman A, Beeson PB (1949). Influence of various disease states upon the febrile response to intravenous injection of typhoid bacterial pyrogen; with particular reference to malaria and cirrhosis of the liver. J Lab Clin Med.

[B29] Rubenstein M, Mulholland JH, Jeffery GM, Wolff SM (1965). Malaria Induced Endotoxin Tolerance. Proc Soc Exp Biol Med.

[B30] Sehgal VM, Siddjiqui WA, Alpers MP (1989). A seroepidemiological study to evaluate the role of passive maternal immunity to malaria in infants. Trans R Soc Trop Med Hyg.

[B31] Desowitz RS (1988). Prenatal immune priming in malaria: antigen-specific blastogenesis of cord blood lymphocytes from neonates born in a setting of holoendemic malaria. Ann Trop Med Parasitol.

[B32] Garner PA (1989). The epidemiology of maternal and neonatal health in Papua New Guinea. MD thesis.

[B33] Vounatsou P, Smith T, Kitua AY, Alonso PL, Tanner M (2000). Apparent tolerance of Plasmodium falciparum in infants in a highly endemic area. Parasitology.

