# Supplementary material for: Three different Plasmodium species show similar patterns of clinical tolerance of malaria infection
Source: Malar J. 2009 Jul 14;8:158. doi: 10.1186/1475-2875-8-158 (PMC2719654; doi:10.1186/1475-2875-8-158)
Supplement: Additional file 1 — Operational characteristics of parasite cut-offs. The additional figure gives age specific values of A: Attributable fractions; B: Sensitivities; C: Specificities for each of the three Plasmodium species. [file 1475-2875-8-158-S1.doc]

# Additional files

**Supplementary Figure 1 - Operational characteristics of parasite cut-offs**

A: Attributable fractions

B: Sensitivities

C: Specificities
